# Developing the DSSAT-CERES-Millet Model for Dynamic Simulation of Grain Protein and Starch Accumulation in Foxtail Millet (*Setaria italica*) Under Varying Irrigation and Nitrogen Regimes

**DOI:** 10.3390/plants14060910

**Published:** 2025-03-14

**Authors:** Shiwei Zhou, Zijin Liu, Fu Chen

**Affiliations:** 1 College of Agronomy and Biotechnology, China Agricultural University, Beijing 100193, China; zhoushiwei@cau.edu.cn (S.Z.); b20243010014@cau.edu.cn (Z.L.); 2 Key Laboratory of Farming System, Ministry of Agriculture and Rural Affairs of China, Beijing 100193, China

**Keywords:** foxtail millet, grain quality, crop growth model, nutritional component simulation, Michaelis–Menten equation, precision agriculture

## Abstract

Foxtail millet (*Setaria italica*), vital in northern China, has its quality and taste influenced by starch and protein. Existing models do not simulate the accumulation of these components during growth. To address this, we enhanced the DSSAT-CERES-Millet model (referred to as DSSAT) by integrating two newly developed modules: the protein simulation module and the starch simulation module. The protein simulation module uses a nitrogen-to-protein conversion coefficient to determine grain protein accumulation based on grain nitrogen accumulation simulated by the DSSAT model. In the starch simulation module, the carbon source supply (carbohydrates) received by millet grains is calculated based on the simulated aboveground and vegetative dry matter by the DSSAT model, and starch synthesis is modeled using the Michaelis–Menten equation to convert carbohydrates into starch within the grains. The integrated model demonstrates good performance in simulating grain protein and starch accumulation, with NRMSE (normalized root mean square error) values of 3.06–26.22% and 4.06–26.88%, respectively. It also accurately simulates grain amylopectin and amylose accumulation at maturity, achieving an NRMSE of less than 14%. The enhanced DSSAT-CERES-Millet model can provide guidance for optimizing irrigation and nitrogen management to enhance the protein and starch quality of millet grains.

## 1. Introduction

Foxtail millet (*Setaria italica*, hereinafter referred to as millet) is an important food crop in northern China, known for its high nutritional value [1]. It is characterized by strong drought resistance and tolerance to poor soil conditions [2]. Millet plays a crucial role in promoting agricultural development in arid regions and balancing the dietary structure of residents [3]. For a considerable period, the primary objective of millet production was to achieve high yields. However, with the enhancement of living standards and the evolution of dietary preferences, consumers now demand superior quality in millet grains. This shift imposes more stringent quality requirements on millet production. Consequently, achieving high quality has emerged as another pivotal goal in contemporary millet production [2]. The protein and starch content in millet grains, which are pivotal traits that critically impact their nutrition, taste, and processing quality, should receive greater attention in millet production [3].

Water and nitrogen are critical factors influencing the quality of millet, and implementing scientific and efficient management practices for water and nitrogen is a common approach to enhancing millet quality [4,5,6,7]. The determination of optimal water and nitrogen management usually relies on field experiments. However, field experiments are often time-consuming and labor-intensive, making it challenging to conduct them at multiple sites and over long periods, which can limit the spatial and temporal applicability of their results [8,9]. By contrast, crop simulation models, which take into account weather-related risks and seasonal variability, are low-cost and efficient tools for determining optimal water and nitrogen management [9,10]. Numerous cropping system models for millet, such as DSSAT [11], AquaCrop [12], and APSIM [13], have been developed. However, these models are typically limited to simulating yield and do not address the simulation of grain quality, including starch and protein. The formation of starch and protein in millet grains during the growth process is influenced by various factors, including the genetic characteristics of the variety, environmental conditions, and cultivation practices [14,15,16,17]. Simulating these processes poses significant challenges, especially for starch [18,19,20,21]. The lack of models for dynamically simulating the accumulation of grain protein and starch is a critical factor limiting the enhancement of millet grain quality through improved water and nitrogen management.

Unlike millet, few models simulating the protein and starch accumulation in the grains of wheat, corn, and rice have been reported [18,20,22,23,24,25,26]. In the existing models, they can roughly be divided into two categories: statistical models based on the effects of ecological factors and mechanistic models based on processes. For the aforementioned statistical models, most are based on ecological factors such as temperature, light, and precipitation after anthesis to predict the final starch and protein accumulation of the grains [24,25]. These models typically have a strong empirical basis, with limited applicability and uncertain precision. Compared to the statistical models, process-based mechanistic models have a higher accuracy in simulating the protein and starch accumulation of grains at maturity [20]. These mechanistic models simulate the accumulation of starch and protein in the grains based on the simulation of the plant carbon and nitrogen dynamics [18,19,20,22]. In these mechanistic models, the rate of starch accumulation in individual grains is determined by the level of carbon source supply and the capacity for assimilate translocation, and grain protein accumulation is the product of the grain nitrogen accumulation and the nitrogen-to-protein conversion factor (typically ranging from 5.18 to 6.25) [18,22,26]. The methods and techniques used to simulate protein and starch accumulation in wheat, corn, and rice grains can serve as a valuable reference for developing models to estimate these components in millet grains.

Some models specifically designed for millet, although lacking the capability to dynamically simulate grain protein and starch accumulation, have been validated for their ability to model the carbon and nitrogen dynamics within the plant, such as DSSAT-CERES-Millet [27,28,29]. The DSSAT-CERES-Millet model is able to accurately simulate growth, development, and yield of millet under various water and nitrogen management strategies [11,30,31,32]. Moreover, the model outputs include plant water and nitrogen stress coefficients, grain number, grain weight, and grain nitrogen accumulation, among others [28]. These features of the DSSAT-CERES-Millet model make it feasible to develop a model for simulating grain protein and starch accumulation in millet on this foundation. In our project, the main objectives were to (1) evaluate the performance of the DSSAT-CERES-Millet model in simulating the carbon and nitrogen dynamics of millet plants, with a focus on the supply of carbon and nitrogen sources to the grains; (2) develop the DSSAT-CERES-Millet model for dynamically simulating grain protein and starch accumulation in millet under varying irrigation and nitrogen regimes.

## 2. Materials and Methods

### 2.1. Experimental Site

A field experiment was performed from 2021 to 2023 at the Organic Dryland Agriculture Experiment Station of China Agricultural University (Shanxi Province, China, 39°88′ N, 113°50′ E). The experimental site has the climate characteristics of warm moist summers (June to August) and cool dry winters (December to February of the following year), with an annual mean minimum temperature of 1.0 ± 0.6 °C, maximum temperature of 14.3 ± 0.7 °C, and precipitation of 389.8 ± 78.6 mm. Precipitation is mainly concentrated from June to September during the summer and autumn, accounting for 70% to 80% of the annual precipitation. There are 0.73 g kg^−1^ available N, 8.02 mg kg^−1^ available P, 19 mg kg^−1^ available K, and 8.70 g kg^−1^ soil organic matter within the upper 20 cm of the soil layer. The soil texture and physical properties for the 0–1 m soil profile at the study site were measured and are presented in Table 1.

### 2.2. Field Experiments

#### 2.2.1. Experimental Design

The millet cultivar used in the experiment was Changza no. 2 (*Setaria italica*), which has been gaining popularity in North China. Sowing and harvesting occurred on 1 May and 3 October, respectively, in 2021; 5 May and 29 September, respectively, in 2022; 2 May and 1 October, respectively, in 2023. The sowing mode adopted hole sowing with a row spacing of 40 cm, and ten to fifteen seeds were manually sown in one seeding hole at a depth of 4 cm. Seedlings were thinned during the 3- to 4-leaf stage, resulting in a planting density of approximately 400,000 plants per ha^−1^. The field experimental treatments included two control factors: irrigation strategy and nitrogen application rate. There were four irrigation strategy options abbreviated as PSI, PVI, RF, and FI. In PSI, a pre-sowing irrigation was adopted, but millet did not receive any irrigation during the growing season. In PVI, irrigation was conducted before sowing and at the vegetative growth stage (emergence–anthesis) during the growing season to avoid water stress on millet before anthesis. RF stands for rain-fed treatment, where no irrigation is applied during the growing period of the millet. In FI, in addition to pre-sowing irrigation, irrigation is also conducted during the whole growing season to avoid water stress on millet. The nitrogen application rate options included four levels: 0, 90, 180, and 270 kg N per ha^−1^. The irrigation strategy options and nitrogen application rates were combined to form the experimental treatments, as detailed in Table 2. Each treatment was repeated three times with each plot sized 10 m (long) × 5 m (wide) in all years. These plots were randomly arranged and separated from each other using 0.8 m wide protective rows.

The irrigation timings and amounts for the PSI, FI, and PVI strategies were based on local farmers’ experience. Pre-sowing irrigation was applied about one week before sowing at 100 mm to raise the soil water content in the 0–1 m layer to field capacity. For in-season irrigation under PVI and FI, when millet leaves curled at midday (indicating water stress), 60 mm of water was applied the next day. Pre-sowing irrigation was applied using border irrigation, while surface drip irrigation was used for all in-season irrigation events. The irrigation events that occurred in all the irrigation treatments were recorded and are presented in Table 2. In each treatment, the full amount of nitrogen fertilizer was applied prior to sowing. Furthermore, all the treatments over the three-year period received consistent rates of phosphorus and potassium fertilizers, also applied pre-sowing. The application rates for phosphorus and potassium fertilizers were set at 240 kg/ha^−1^ (P_2_O_5_) and 72 kg/ha^−1^ (K_2_O), respectively, ensuring adequate nutrient supply to prevent phosphorus and potassium deficiency in the millet. Pest, insect, and weed control measures were carried out following the established practices used by local farmers.

#### 2.2.2. Measurements

Throughout the millet growing seasons across all years, crop phenology was monitored on a daily basis and classified according to the criteria established by Lancashire et al. [33]. The onset and completion times of six critical growth stages were documented: seedling stage, jointing stage, booting stage, heading stage, grain filling stage, and maturity. At each growth stage, leaf area, aboveground biomass, and plant nitrogen accumulation were measured once per plot. Five millet plants, randomly selected from the inner rows and tagged at the seedling stage, were used for nondestructive leaf area measurements using a leaf area meter (Model YMJ-B, Zhejiang Top Cloud-Agri Technology Co., Ltd., Hangzhou, China). The leaf area index (LAI) was estimated as a ratio of the green leaf area to unit ground area. To measure aboveground biomass and plant nitrogen accumulation, ten plants per plot were sampled. Each plant was cut at the ground surface and separated into stems, leaves, and grains. These parts were dried in an oven at 105 °C for 30 min, then at 75 °C until constant weight. The dried parts were ground (<1 mm) for nitrogen content analysis using the Kjeldahl method [34]. Plant nitrogen accumulation was calculated by summing the nitrogen accumulation of each part, determined by multiplying the dry weight by the nitrogen concentration.

Particular focus was placed on the process of grain formation following anthesis. Spike samples were collected at intervals of 7 to 10 days, starting from anthesis and continuing until maturity. These samples were meticulously analyzed to determine key grain characteristics, including grain weight, grain nitrogen accumulation, grain protein accumulation, grain starch accumulation, grain amylose accumulation, and grain amylopectin accumulation. For each sampling, 10 spikes were collected from each plot and immediately frozen in liquid nitrogen. From the central part of these spikes, 100 grains were selected and stored at −80 °C until analysis. Grain nitrogen accumulation was determined using the Kjeldahl method, and grain protein accumulation was measured with a transmissive near-infrared spectrometer (Infraneo Junior, Chopin Technologies, Paris, France) [35]. The concentration of grain starch (including amylose and amylopectin) was determined using a coupled spectrophotometer [36]. At the stage of physiological maturity, the ears of all millet plants in each plot were harvested and threshed, followed by natural drying to determine grain yield. Moreover, grain nitrogen accumulation, grain protein accumulation, grain starch accumulation, grain amylose accumulation, and grain amylopectin accumulation were also measured at maturity. Grain yield was standardized to 14% moisture. Note that the LAI, aboveground biomass, phenology, and grain yield were measured across all three years, while such parameters as plant nitrogen accumulation, grain nitrogen accumulation, grain protein accumulation, grain starch accumulation, grain amylose accumulation, and grain amylopectin accumulation were measured only in 2022 and 2023.

### 2.3. Models and Methods for Simulating Grain Protein and Starch Accumulation

**Figure 1:**
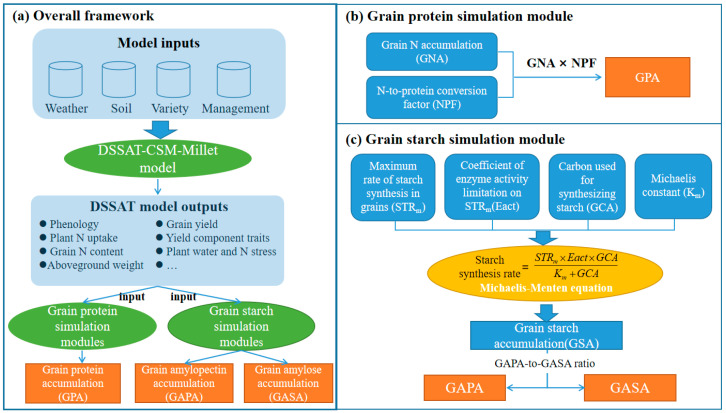
Overall framework of the joint model and its key modules for dynamically simulating grain protein and starch accumulation in foxtail millet.

In this study, the simulation of millet grain protein and starch accumulation was conducted using the DSSAT-CSM-Millet model, enhanced with specific modules for starch and protein simulation. The protein and starch accumulation of millet grains were simulated using the protein simulation module and the starch simulation module, respectively. The outputs from the DSSAT-CSM-Millet model provided data support for both modules. The overall framework of the joint model and its key modules for simulating grain protein and starch accumulation is shown in Figure 1.

#### 2.3.1. Description of DSSAT-CERES-Millet

DSSAT is a widely used process-based crop modeling system that supports agronomic decision-making by simulating crop growth and development, soil water, carbon, and nitrogen processes, as well as management practices [28,29]. Within DSSAT, the CERES series models are primarily used to simulate the growth and development of cereal crops, including millet [29]. In this study, the CERES model for millet (DSSAT-CERES-Millet model, version 4.8) was employed to simulate the accumulation and allocation of carbon and nitrogen within the plant and their transfer to the grains under varying irrigation and nitrogen treatments [28]. Similarly to other CERES models, the DSSAT-CERES-Millet model simulates millet yield, growth, and development based on phenology, photoperiod, and photosynthetically active radiation, while accounting for water and nitrogen stress limitations [27]. The model’s nitrogen uptake mechanism operates on a supply-and-demand basis, where the demand is driven solely by the vegetative components of the plant, including leaves, stems, and roots [27]. The crop’s demand for nitrogen is regulated by a critical nitrogen concentration in the shoots, which is optimal for maximizing crop biomass production and decreases as the plant’s dry matter increases [37]. Nitrogen supply from the soil depends on nutrient availability and the absorption capacity of the roots within each soil layer. The partitioning of absorbed nitrogen between shoots and root parts is determined by the respective demands of these components [27]. The dataset required to drive the model includes daily meteorological data, soil physical and chemical properties, crop genetic parameters, and field management practices. Detailed descriptions of the DSSAT-CERES-Millet model can be found in studies by Jones et al. [29] and Virmani et al. [27].

#### 2.3.2. Description of Grain Protein Simulation Modules

The grain protein accumulation of millet was calculated by multiplying the grain nitrogen accumulation with a nitrogen-to-protein conversion factor (NPF). The NPF was affected by water stress and nitrogen stress [26]. The grain protein accumulation was estimated as follows:

(1) $$GPA_{i}=GNA_{i}\times NPF_{i},$$

(2) $$NPF_{i}=\delta \times MIN\;[f(W_{i}),f(N_{i})]+NPF_{0},$$

where *GPA_i_* (kg/ha^−1^) is the grain protein accumulation, *GNA_i_* (kg/ha^−1^) is the grain nitrogen accumulation on the *i*th day after anthesis, *NPF*_0_ is the baseline value for NPF, *δ* is the correction factor for *NPF*_0_, and *f*(*W_i_*) and *f*(*N_i_*) represent the water and nitrogen stress levels of the plant on the *i*th day after anthesis, respectively, where *f*(*W_i_*) is calculated as the ratio of the actual evapotranspiration (*Ta_i_*) to the potential evapotranspiration (*Tp_i_*) and *f*(*N_i_*) is determined by the plant nitrogen concentration.

(3) $$f(W_{i})=Ta_{i}/Tp_{i},$$

(4) $$f(N_{i})=MIN(1,\;\;\frac{ANP_{i}-MNP}{CNP-MNP}),$$

where *ANP_i_* is the plant nitrogen concentration on the *i*th day after anthesis, *CNP* is the critical plant nitrogen concentration, and *MNP* is the minimum plant nitrogen concentration. The values of *GNA_i_*, *f*(*W_i_*), and *f*(*N_i_*) are determined by the output values of the DSSAT-CERES-Millet model.

#### 2.3.3. Description of Grain Starch Simulation Modules

The dynamic process of starch accumulation in millet grains is calculated as follows:

(5) $$GSA_{i}=SP\times Ng\times IGSA_{i}\times 10^{-6},$$

(6) $$IGSA_{i}=\left\{\begin{array}{cc} IGSA_{0}+ISTR_{1} & i=1\\ IGSA_{i-1}+ISTR_{i} & i>1 \end{array}\right.,$$

where *GSA_i_* (kg/ha^−1^) represents the accumulated starch amount on the *i*th day after anthesis and *SP* (spikes/ha^−1^) and *Ng* (grains/spike^−1^) represent the number of panicles and the number of grains, respectively. The values of *SP* and *Ng* are obtained from the outputs of the DSSAT-CERES-Millet model; *IGSA_i_* (mg per grain) and *ISTR_i_* (mg/day^−1^ per grain) represent the accumulation amount and accumulation rate of starch in individual grains, respectively, on the *i*th day after anthesis, and *IGSA*_0_ (mg per grain) is the initial starch content in individual grains.

According to Ji et al. [18] and Chen [23], *ISTR_i_* is constrained by two factors: one is the amount of available carbon for starch synthesis in the grains during the grain filling period, and the other is the starch synthesis capacity of the millet. The capacity for starch synthesis in grains is influenced by both the genetic characteristics of the variety and external environmental conditions, with the activity of related enzymes being an intuitive manifestation of this process [23]. This study utilized the Michaelis–Menten equation to model the process of starch synthesis from carbon sources, specifically sucrose, within grains. The Michaelis–Menten equation is a classic formula in enzyme kinetics that describes the relationship between the rate of enzyme-catalyzed reactions and substrate concentration [38].

(7) $$ISTR_{i}=\frac{ISTR_{m}\times Eact_{i}\times GCA_{i}}{K_{m}+GCA_{i}},$$

where *ISTR_m_* (mg/day^−1^ per grain) represents the maximum rate of grain starch accumulation, which varies between cultivars and is considered a genotype parameter in the model; *Eact_i_* represents the limiting factor of enzyme activity related to starch synthesis and the rate of starch synthesis, with values ranging from 0 to 1; *K_m_* (mg per grain) represents the Michaelis constant; *GCA_i_* (mg per grain) represents the carbon source available for starch synthesis in the grains on the *i*th day after anthesis.

As reported by Ji et al. [18], the grains of millet receive carbon sources in two ways: one part comes from immediate photosynthetic products, while the other part is derived from non-structural carbohydrates (primarily soluble sugars) transported by nutritional organs such as stems and leaves. The immediate photosynthetic products received by the grains can be determined by the dynamic changes in the total aboveground dry weight of the plant and the total dry weight of its vegetative organs following anthesis. The photosynthetic products transferred from vegetative organs to the grains can be calculated based on the dynamic changes in the dry weight of the vegetative organs after anthesis. *GCA_i_* is calculated based on the following equations [18].

(8) $$GCA_{i}=10^{6}\times (GCP_{i}+GCT_{i})/(SP\times Ng),$$

(9) $$GCP_{i}=\left\{\begin{array}{cc} \left(TOPWT_{i}-TOPWT_{i-1}\right)-\left(VWT_{i}-VWT_{i-1}\right) & GDD_{i}<GDD_{m}\\ TOPWT_{i}-TOPWT_{i-1} & GDD_{i}\geq GDD_{m} \end{array}\right.,$$

(10) $$GCT_{i}=\left\{\begin{array}{cc} 0 & GDD_{i}<GDD_{m}\\ VWT_{i-1}-VWT_{i} & GDD_{i}\geq GDD_{m} \end{array}\right.,$$

where *GCP_i_* (kg/ha^−1^) represents the immediate photosynthetic products received by the grains, *GCT_i_* (kg/ha^−1^) represents the photosynthetic products transferred from vegetative organs to the grains, *TOPWT_i_* (kg/ha^−1^) represents the aboveground dry weight, and *VWT_i_* (kg/ha^−1^) represents the dry weight of the vegetative organs on the *i*th day after anthesis; *GDD_i_* (°C d) refers to the accumulated temperature within *i* days after anthesis; and *GDD_m_* (°C d) is a time scale that measures the accumulated temperature from the stage of anthesis to the point when the activity of synthetic amylase in grains reaches its peak level. Specifically, values exceeding this metric indicate the initiation of assimilate remobilization and a subsequent decrease in the weight of vegetative organs. The values of *TOPWT_i_* and *VWT_i_* are obtained from the outputs produced by the DSSAT-CERES-Millet model.

In the calculation of Eacti, the effects of external environmental factors (air temperature and water and nitrogen supply) and the plant lifecycle on the activity of starch synthesis-related enzymes are considered. The calculation formula is as follows [23]:

(11) $$Eact_{i}=f(GDD_{i})\times f(T_{i})\times f(W_{i})\times f(N_{i}),$$

where *f*(*GDD_i_*), *f*(*T_i_*), *f*(*W_i_*), and *f*(*N_i_*), respectively, represent the effects of the plant lifecycle, temperature, plant water status, and plant nitrogen status on the activity of starch synthesis-related enzymes; the values of these functions range from 0 to 1.

It is widely believed that the activity of enzymes related to starch synthesis after anthesis initially increases and then decreases over time [18,39,40]. Specifically, enzyme activity exhibits an exponential increase with accumulating temperature after anthesis, followed by a linear decline [18]. The corresponding function is given by the following equation:

(12) $$f(GDD_{i})=\left\{\begin{array}{ll} \frac{GDD_{i}}{GDD_{m}}\cdot \exp[\gamma (GDD_{m}-GDD_{i})] & GDD_{i}\leq GDD_{m}\\ \frac{GDD_{\mathrm{AM}}-GDD_{i}}{GDD_{\mathrm{AM}}-GDD_{m}} & GDD_{i}>GDD_{m} \end{array}\right.,$$

where *GDD_AM_* (°C d), *GDD_m_* (°C d), and *γ* are module parameters and vary with different millet varieties. *GDD_i_* and *GDD_m_* are defined in Equations (9) and (10); *γ* represents the sensitivity coefficient of amylase activity to the accumulation of growing degree days after anthesis. *GDD_AM_* represents the accumulated temperature required for millet from anthesis to maturity, which is calculated based on the simulated phenology using the DSSAT-CERES-Millet model and meteorological data.

Temperature has a remarkably significant impact on the activity of starch synthase. Specifically, there is a specific optimal temperature at which enzyme activity can reach its peak. When the temperature gradually increases within a certain range, the activity of the enzyme will be enhanced accordingly. However, once the temperature exceeds the appropriate range and becomes too high, it will cause the enzyme to undergo denaturation and lose its activity. Conversely, when the temperature drops, the activity of the enzyme will also decline. The following equation is used to calculate *f*(*T_i_*) [23]:

(13) $$f(T_{i})=\left\{\begin{array}{cc} \sin\left[\frac{T_{i}-T_{b}}{T_{ol}-T_{b}}\times \frac{\pi }{2}\right] & T_{b}\leq T_{i}<T_{ol}\\ 1 & T_{ol}\leq T_{i}<T_{oh}\\ \sin\left[\frac{T_{m}-T_{i}}{T_{m}-T_{oh}}\times \frac{\pi }{2}\right] & T_{oh}\leq T_{i}<T_{m}\\ 0 & T_{m}<T_{i}\;or\;T_{i}<T_{b} \end{array}\right.,$$

where *T_i_* (°C) is the daily average temperature on the *i*th day after anthesis; *T_ol_* and *T_oh_* are the lower and upper limit values (°C) of the optimal temperature for grain starch formation, respectively; and *T_b_* and *T_m_* are the minimum and maximum temperatures required for grain starch formation, respectively. Taking into account the close relationship between grain filling and starch formation, we leveraged this connection to establish relevant indicators. For millet, based on the settings from the DSSAT-CERES-Millet model regarding the impact of temperature on grain filling, the values for *T_ol_* and *T_oh_* are 22 °C and 27 °C, respectively, while *T_b_* and *T_m_* are 7 °C and 60 °C, respectively [28].

The accumulation amounts of amylopectin and amylose in millet grains are calculated based on grain starch accumulation and the proportions of these two components within grain starch accumulation as follows:

(14) $$GASA_{i}=GSA_{i}\times RA_{i},$$

(15) $$GAPA_{i}=GSA_{i}\times (1-RA_{i}),$$

where *GASA_i_* (kg/ha^−1^) represents the grain amylose accumulation, *GAPA_i_* (kg ha^−1^) represents the grain amylopectin accumulation, and *RA_i_* (%) represents the ratio of amylose accumulation to the total starch accumulation in the grains on the *i*th day after anthesis. *RA_i_* is calculated using the following equation [23]:

(16) $$RA_{i}=(\alpha \times \ln(GDD_{i})-\beta )\times f(N_{i}),$$

where *α* is a scaling factor that determines how the natural logarithm of growing degree days (ln(*GDD_i_*)) influences the ratio of amylose accumulation relative to total starch; *β* is a correction factor that adjusts the baseline ratio of amylose to total starch independent of heat accumulation and nitrogen stress effects. Equation (4) is used to calculate *f*(*N_i_*).

#### 2.3.4. Model Calibration, Validation, and Evaluation

In this study, the model calibration and validation process was divided into three primary components. The first component involved the calibration and validation of parameters for the DSSAT-CERES-Millet model, specifically focusing on seven genetic coefficients (P1, P20, P2R, P5, G1, G4, and PHINT), which are crucial for accurately simulating millet growth and development. The second component centered on the calibration and validation of the grain protein simulation module, with key parameters including *δ* and *NPF*_0_ being calibrated to ensure precise modeling of grain protein accumulation. The third component focused on the calibration and validation of the grain starch simulation module, where key parameters such as *GDD_m_*, *γ*, *IGST*_0_, *ISTR_m_*, *K_m_*, *α*, and *β* were calibrated to ensure accurate modeling of grain starch attributes.

The field experimental datasets from all the treatments in 2021 were used for the calibration of genetic parameters in the DSSAT-CERES-Millet model, while the data from 2022 and 2023 were utilized for model validation. These datasets included phenology (anthesis and maturity), LAI, aboveground biomass, plant nitrogen accumulation, grain nitrogen accumulation, and grain yield. The calibration process employed the Generalized Likelihood Uncertainty Estimation (GLUE) submodule within DSSAT to adjust the genetic coefficients, aiming to minimize the difference between the simulated and measured data, such as phenology and millet yield. GLUE is a Bayesian estimation method that determines the probability distribution between observed data and those estimated by the model [41]. This process involved two rounds, each consisting of 3000 iterations. The first round focused on adjusting the phenology parameters to accurately simulate key growth stages, while the second round concentrated on estimating the growth parameters essential for simulating the LAI, aboveground biomass, and grain yield. Considering the DSSAT-CERES-Millet model performance in simulating plant nitrogen accumulation and grain nitrogen accumulation, the calibration results of genetic coefficients from the GLUE were corrected using the trial-and-error method.

For the grain protein and starch simulation modules, the field experimental datasets from all the treatments in 2022 and 2023 were used for module calibration and validation, respectively, including grain protein accumulation, grain starch accumulation, grain amylopectin accumulation, and grain amylose accumulation. After calibrating the DSSAT-CERES-Millet model, parameters *δ* and *NPF*_0_ were adjusted to minimize the root mean square error (RMSE) between the simulated and observed grain protein accumulation. Similarly, parameters *IGST*_0_, *ISTR_m_*, *K_m_*, *GDD_m_*, and *γ* were fine-tuned to achieve the lowest RMSE between the simulated and observed grain starch accumulation. Additionally, parameters *α* and *β* were optimized to minimize the RMSE between the simulated and observed grain amylopectin accumulation and grain amylose accumulation. The optimization of the aforementioned parameters was performed using a genetic algorithm, which was implemented using the GA package (version 3.2.4) in R. The GA package provides robust tools specifically designed for solving complex optimization problems through genetic algorithms [42]. The calculation of the RMSE can be found in Equation (18) below. The final calibration results of these model or module parameters are presented in Table 3.

In both model calibration and validation, the performance of each model or module was quantified using the mean relative difference (MRD), the RMSE, and the normalized RMSE (NRMSE). They were computed as follows:

(17) $$MRD=\frac{1}{n}\sum_{i=1}^{n}\frac{\left(P_{i}-O_{i}\right)}{O_{i}}\times 100\%,$$

(18) $$RMSE=\sqrt{\frac{1}{n}\sum_{i=1}^{n}{\left(P_{i}-O_{i}\right)}^{2}},$$

(19) $$NRMSE=\frac{RMSE}{O_{avg}}\times 100\%$$

where, *P_i_* and *O_i_* are the simulated and observed values, respectively, for the *i*th data pair; *O_avg_* is the average of the observed values; and *n* is the number of observed or simulated datapoints. Based on the NRMSE value, model performance can be classified into four categories: excellent (NRMSE < 10%), good (10% ≤ NRMSE < 20%), moderate (20% ≤ NRMSE < 30%), and poor (NRMSE ≥ 30%) [43].

## 3. Results

### 3.1. Performance of the DSSAT-CERES-Millet Model

**Figure 2:**
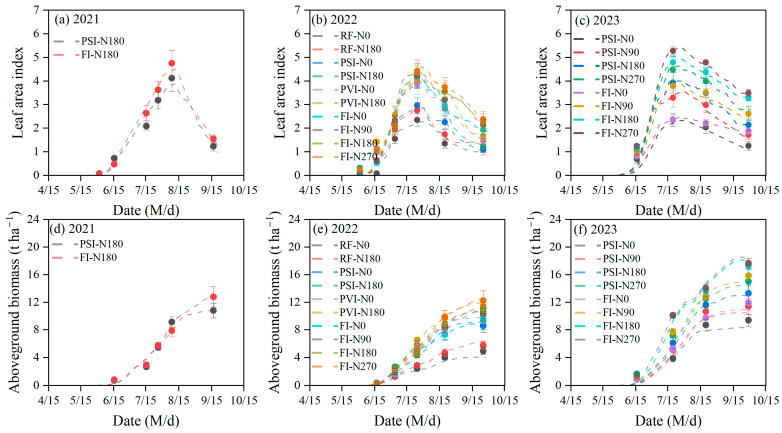
Comparison between the measured leaf area index (**a**–**c**) and aboveground biomass (**d**–**f**) of foxtail millet and the simulated values from the DSSAT-CERES-Millet model under different water and nitrogen treatments from 2021 to 2023. Note: The scatter points in the graph denote the observed values for each experimental treatment as represented by the curves of corresponding colors. RF, PSI, PVI, and FI indicate different irrigation strategies. RF represents no irrigation or rain-fed treatment. In PSI, only a pre-sowing irrigation is adopted. In PVI, irrigation is conducted before sowing and at the vegetative growth stage of foxtail millet. In FI, irrigation is conducted before sowing and during the growing season to avoid water stress on foxtail millet. N0, N90, N180, and N270 are nitrogen application rates of 0, 90, 180, and 270 kg/ha^−1^, respectively. Error bars indicate the standard deviation.

The DSSAT-CERES-Millet model effectively simulates the dynamic changes in the LAI and aboveground biomass of millet under various water and nitrogen treatments with a relatively high accuracy (Figure 2 and Table 4). From 2021 to 2023, the model’s simulation errors for the LAI and aboveground biomass under different water and nitrogen conditions exhibited the RMSE, the NRMSE, and the MRD values ranging from 0.19 to 0.42, from 8.27% to 18.35%, and from −25.20% to 45.80% for the LAI, respectively, and from 0.10 to 1.27 t/ha^−1^, from 1.65% to 15.07%, and from −28.92% to 9.60% for the aboveground biomass, respectively. The model slightly underestimates the LAI, a common occurrence when modeling various water and nitrogen treatments, and accurately captures the inhibitory effects of water and nitrogen deficits on the leaf area growth and dry matter production. Specifically, the simulated LAI and aboveground biomass were lower under reduced water and nitrogen conditions, consistent with observational data. The highest errors (NRMSE) in simulating the LAI and aboveground biomass occurred under the RF-N0 treatment, which had the smallest irrigation and nitrogen application rates, indicating some uncertainty in the model’s performance under water and nitrogen deficits. Additionally, we observed that the model’s simulation errors (NRMSE) for the LAI and aboveground biomass tended to increase under reduced irrigation within the same nitrogen application levels. Similarly, there was a higher NRMSE under reduced nitrogen treatments within the same irrigation levels. Overall, the model faces challenges in simulating the LAI and aboveground biomass under conditions of low water or nitrogen supply.

**Figure 3:**
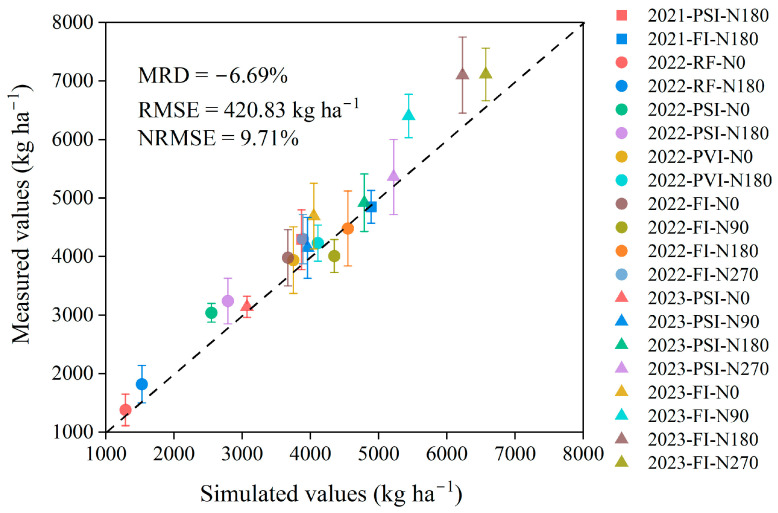
Comparison between the measured grain yield of foxtail millet and the simulated values from the DSSAT-CERES-Millet model under the different water and nitrogen treatments from 2021 to 2023. Note: RF, PSI, PVI, and FI indicate different irrigation strategies. RF represents no irrigation or rain-fed treatment. In PSI, only a pre-sowing irrigation is adopted. In PVI, irrigation is conducted before sowing and at the vegetative growth stage of foxtail millet. In FI, irrigation is conducted before sowing and during the growing season to avoid water stress on foxtail millet. N0, N90, N180, and N270 are nitrogen application rates of 0, 90, 180, and 270 kg/ha^−1^, respectively. Error bars indicate the standard deviation. RMSE, NRMSE, and MRD stand for the root mean square error, the normalized RMSE, and the mean relative difference, respectively.

The DSSAT-CERES-Millet model provides accurate simulations of millet phenology, with differences between the simulated and observed dates for anthesis and maturity within 5 days across all the water and nitrogen treatments from 2021 to 2023 (Table 5). The observational data indicate that water and nitrogen management have a minimal effect on millet phenology. Within the same year, the variations in anthesis and maturity timing across the different water and nitrogen treatments were less than 7 days. Notably, the simulated phenology for both anthesis and maturity remained consistent across different treatments in each year. For millet yield simulation, the model also demonstrated notable performance with an RMSE of 420.83 kg/ha^−1^ and an NRMSE of 9.71%, but it generally underestimated the yield (MRD of −6.69%) (Figure 3). The largest deviation occurred in the PSI-N0 treatment in 2022 with low irrigation and nitrogen application rates, where the predicted yield deviated by −16.1% from the observed value. The model not only showed higher simulation errors in yield under conditions of low water and nitrogen supply, but also exhibited notable uncertainty when simulating yield under high water and nitrogen supply treatments. Treatments FI-N90 and FI-N180 from 2023 also exhibited notable simulation biases, with MRDs of −15.0% and −13.7%, respectively.

**Figure 4:**
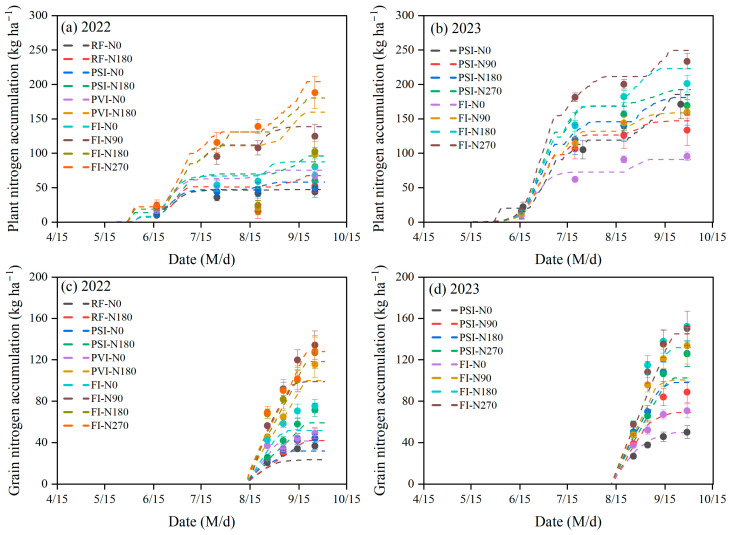
Comparison between the measured plant nitrogen accumulation (**a**,**b**) and grain nitrogen accumulation (**c**,**d**) of foxtail millet and the simulated values from the DSSAT-CERES-Millet model under the different water and nitrogen treatments in 2022 and 2023. Note: The scatter points in the graph denote the observed values for each experimental treatment as represented by the curves of corresponding colors. RF, PSI, PVI, and FI indicate different irrigation strategies. RF represents no irrigation or rain-fed treatment. In PSI, only a pre-sowing irrigation is adopted. In PVI, irrigation is conducted before sowing and at the vegetative growth stage of foxtail millet. In FI, irrigation is conducted before sowing and during the growing season to avoid water stress on foxtail millet. N0, N90, N180, and N270 are nitrogen application rates of 0, 90, 180, and 270 kg/ha^−1^, respectively. Error bars indicate the standard deviation.

Overall, the DSSAT-CERES-Millet model performs well in capturing both the dynamic trends and the accuracy of nitrogen accumulation in both plants and grains (Table 6 and Figure 4). The model tends to overestimate plant nitrogen accumulation while underestimating grain nitrogen accumulation. For plant nitrogen accumulation under various water and nitrogen treatments, the model showed a high accuracy, with the MRD ranging from −5.47% to 14.55%, the RMSE—from 4.98 to 16.58 kg/ha^−1^, and the NRMSE—from 6.19% to 18.66%. In contrast, the model’s accuracy in simulating grain nitrogen accumulation was generally lower, with the MRD from −29.75% to 13.96%, the RMSE—from 6.81 to 24.59 kg/ha^−1^, and the NRMSE—from 10.02% to 32.39%. Notably, except for the RF-N0 treatment in 2022, the NRMSE for grain nitrogen accumulation in all the other treatments was below 30%. Generally, the simulation errors for nitrogen accumulation in plants and grains are higher under low-nitrogen treatments. This phenomenon has been observed in both high-irrigation and low-irrigation treatments. For high-nitrogen treatments, the simulation errors for plant and grain nitrogen accumulation are typically lower under higher irrigation levels at the same nitrogen application rates. The highest NRMSE for plant and grain nitrogen accumulation was observed in the RF-N0 treatment in 2022 among all the water and nitrogen treatments.

### 3.2. Simulation of Grain Protein Accumulation

**Figure 5:**
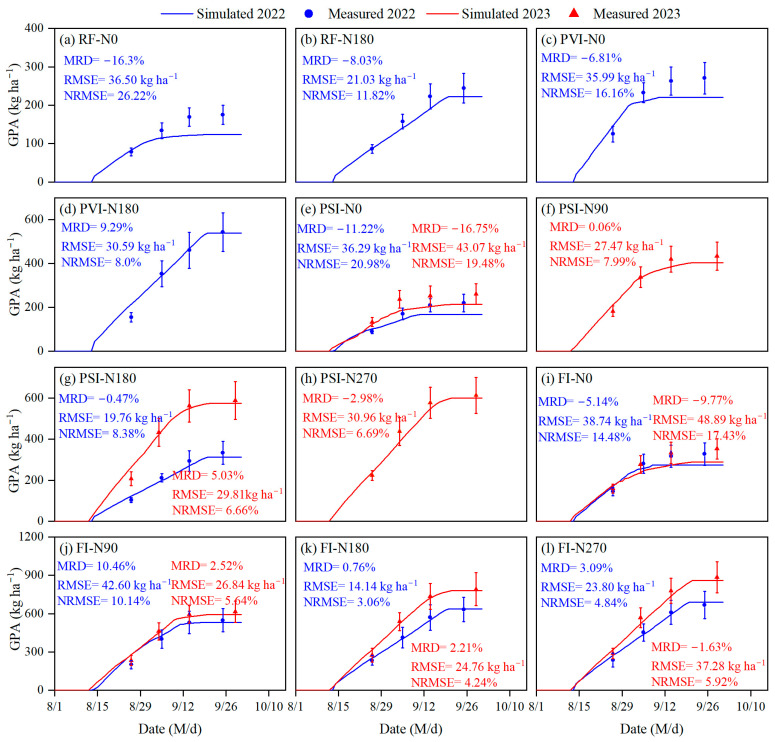
Comparison of the measured grain protein accumulation (GPA) in foxtail millet versus the simulated values from the enhanced DSSAT-CERES-Millet model, incorporating a grain protein simulation module, under the various water and nitrogen treatments in 2022 and 2023. Note: RF, PSI, PVI, and FI indicate different irrigation strategies. RF represents no irrigation or rain-fed treatment. In PSI, only a pre-sowing irrigation is adopted. In PVI, irrigation is conducted before sowing and at the vegetative growth stage of foxtail millet. In FI, irrigation is conducted before sowing and during the growing season to avoid water stress on foxtail millet. N0, N90, N180, and N270 are nitrogen application rates of 0, 90, 180, and 270 kg/ha^−1^, respectively. Error bars indicate the standard deviation. RMSE, NRMSE, and MRD stand for the root mean square error, the normalized RMSE, and the mean relative difference, respectively.

As shown in Figure 5, the simulated and observed values for grain protein accumulation exhibited matching trends, both increasing after anthesis and then stabilizing over time across all the water and nitrogen treatments in 2022 and 2023. Overall, the combined model of the protein simulation module and the DSSAT-CERES-Millet model demonstrated a high level of accuracy in simulating grain protein accumulation for these treatments. For the grain protein accumulation simulations, the RMSE, NRMSE, and MRD were 14.14–48.89 kg/ha^−1^, 3.06–26.22%, and −16.75–10.46%, respectively. The module is prone to underestimating grain protein accumulation under low-irrigation treatments, especially near maturity. The highest NRMSE occurred under the RF-N0 treatment, which represented rain-fed conditions without nitrogen application. Additionally, the PVI-N0, PSI-N0, and FI-N0 treatments, all representing low-nitrogen conditions, exhibited relatively high errors. Specifically, the NRMSE values were 16.16% for PVI-N0, 19.48–20.98% for PSI-N0, and 14.48–17.43% for FI-N0. These results highlight the model’s challenges in accurately simulating grain protein accumulation under such low-nitrogen conditions.

### 3.3. Simulation of Grain Starch Accumulation

**Figure 6:**
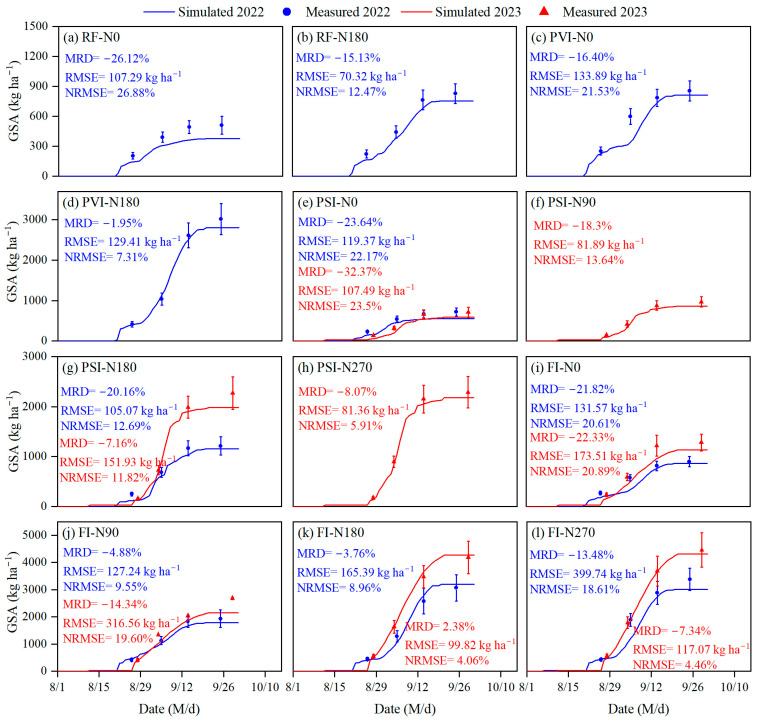
Comparison of the measured grain starch accumulation (GSA) in foxtail millet versus the simulated values from the enhanced DSSAT-CERES-Millet model, incorporating a grain starch simulation module, under the various water and nitrogen treatments in 2022 and 2023. Note: RF, PSI, PVI, and FI indicate different irrigation strategies. RF represents no irrigation or rain-fed treatment. In PSI, only a pre-sowing irrigation is adopted. In PVI, irrigation is conducted before sowing and at the vegetative growth stage of foxtail millet. In FI, irrigation is conducted before sowing and during the growing season to avoid water stress on foxtail millet. N0, N90, N180, and N270 are nitrogen application rates of 0, 90, 180, and 270 kg/ha^−1^, respectively. Error bars indicate the standard deviation. RMSE, NRMSE, and MRD stand for the root mean square error, the normalized RMSE, and the mean relative difference, respectively.

The DSSAT-CERES-Millet model, integrated with the grain starch simulation module, effectively simulates grain starch accumulation under various water and nitrogen treatments, capturing trends accurately and achieving high precision with an RMSE of 70.32–399.74 kg/ha^−1^ and an NRMSE of 4.06–26.88% (Figure 6). However, it is important to note that the model tends to generally underestimate grain starch accumulation across these treatments, as indicated by an MRD from −26.12% to 2.38%. Low-nitrogen treatments generally exhibit higher simulation errors. The NRMSE for grain starch accumulation under the no-nitrogen (N0) treatments, including RF-N0, PSI-N0, PVI-N0, and FI-N0, was markedly higher compared to the other water and nitrogen treatments, with the errors exceeding 20%. Among these treatments, RF-N0 exhibited the highest NRMSE. Under identical nitrogen application rates, the model’s simulations of grain starch accumulation generally exhibited a decline in accuracy at lower irrigation levels compared to higher ones. Overall, simulating grain starch accumulation under conditions of water and nitrogen deficiency is notably more challenging. Notably, the FI-N270 treatment in 2022 exhibited a high NRMSE of 18.61%, despite having a much lower NRMSE of 4.46% in 2023, indicating a notable model uncertainty in simulating grain starch accumulation under conditions of high water and high nitrogen supply.

**Figure 7:**
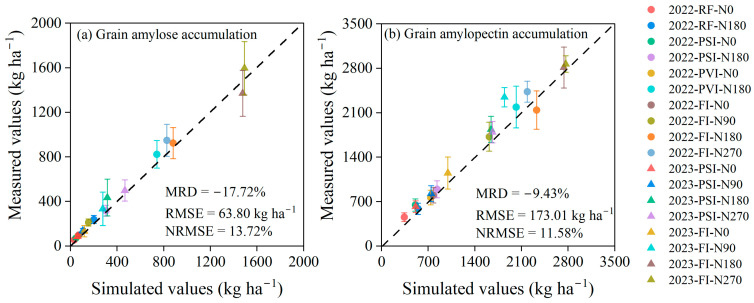
Comparison of the measured grain amylose (**a**) and amylopectin (**b**) accumulation in foxtail millet at maturity versus the simulated values from the enhanced DSSAT-CERES-Millet model, incorporating a grain starch simulation module, under the various water and nitrogen treatments in 2022 and 2023. Note: RF, PSI, PVI, and FI indicate different irrigation strategies. RF represents no irrigation or rain-fed treatment. In PSI, only a pre-sowing irrigation is adopted. In PVI, irrigation is conducted before sowing and at the vegetative growth stage of foxtail millet. In FI, irrigation is conducted before sowing and during the growing season to avoid water stress on foxtail millet. N0, N90, N180, and N270 are nitrogen application rates of 0, 90, 180, and 270 kg/ha^−1^, respectively. Error bars indicate the standard deviation. RMSE, NRMSE, and MRD stand for the root mean square error, the normalized RMSE, and the mean relative difference, respectively.

Moreover, the performance of the enhanced DSSAT-CERES-Millet model in simulating the grain amylose and amylopectin accumulation in millet at maturity was also investigated and are shown in Figure 7. Our findings demonstrated that the enhanced crop model performed well in simulating these starch components under various water and nitrogen treatments, although it frequently exhibited a slight underestimation of the values. Specifically, for grain amylose accumulation simulations, the model achieved the RMSE, NRMSE, and MRD values of 63.80 kg/ha^−1^, 13.72%, and −17.72%, respectively. For grain amylopectin accumulation simulations, the corresponding values were 173.01 kg/ha^−1^, 11.58%, and −9.43%. The enhanced model generally showed larger simulation errors for grain amylose and amylopectin accumulation under conditions of low water and nitrogen availability. Specifically, the largest deviations in simulating grain amylose and amylopectin accumulation occurred under RF-N0 in 2022, with the MRD values of −37.3% and −25.2%, respectively.

## 4. Discussion

### 4.1. Performance of the DSSAT-CERES-Millet Model

In this study, the DSSAT-CERES-Millet model provided an essential foundation for modeling the accumulation of protein and starch within millet grains. The outputs from the DSSAT-CERES-Millet model delivered vital data support that is necessary for accurately simulating the accumulation of protein and starch in the grains (Figure 1). Thus, it was essential to focus on the DSSAT-CERES-Millet model’s capabilities in simulating key aspects of plant nitrogen and carbon dynamics of millet, including phenology, LAI, aboveground biomass, plant nitrogen accumulation, grain nitrogen accumulation, and grain yield. The results of this study demonstrate that the DSSAT-CERES-Millet model achieved an NRMSE ranging from 8.27% to 18.35% in simulating the LAI, from 1.65% to 15.07% for the aboveground biomass, and 9.71% for grain yield simulations under the various water and nitrogen treatments (Table 4 and Figure 3). According to the evaluation criteria set by Jamieson et al. [43], a model’s performance is deemed good when the NRMSE is below 20%. Based on these criteria, our findings suggest that the DSSAT-CERES-Millet model performs commendably in capturing the carbon dynamics within millet plants. This conclusion is also supported by previous studies, which reported similar ranges of the NRMSE values: LAI simulations typically range from 12.9% to 20.6%, aboveground biomass—from 3.5% to 26.9%, and grain yield—from 1.0% to 11.8% [11,31,44]. It should be noted that the DSSAT-CERES-Millet model’s performance in simulating the LAI, aboveground biomass, and grain yield was less accurate under low water and nitrogen conditions compared to higher water and nitrogen conditions (Table 4 and Figure 3). Under low water and nitrogen conditions, crops initiate complex adaptive mechanisms to cope with resource limitations. These mechanisms include physiological and morphological changes such as altered root distribution, adjusted stomatal behavior, and reconfigured metabolic pathways [45,46]. The high complexity and variability of these responses make it more challenging to accurately simulate crop growth under low water and nitrogen conditions. Additionally, we found that the model not only has higher simulation errors in yield under low water and nitrogen conditions, but also shows a notable uncertainty in yield simulations under high water and nitrogen treatments (FI-N90 and FI-N180) (Figure 3). This may be due to the fact that under high water and nitrogen conditions, crops tend to exhibit luxuriant growth, which is challenging for the current CERES model to simulate accurately [27,28,29].

Similar to other CERES models (such as CERES-Wheat, CERES-Maize, etc.), the DSSAT-CERES-Millet model is unable to simulate the effects of water and nitrogen management on phenology (Table 5). This is because the phenology simulation in the current model primarily relies on meteorological factors such as temperature and photoperiod, without accounting for the additional influences of water and nitrogen conditions [27,29]. Nevertheless, the DSSAT-CERES-Millet model demonstrated a simulation bias of less than 5 days for anthesis and maturity. This can be attributed to the relatively minor impact of water and nitrogen on the phenology of millet, as evidenced by the field experiment results (Table 5). Compared to its simulation of the abovementioned growth indicators, the DSSAT-CERES-Millet model showed lower accuracy in simulating plant nitrogen accumulation and grain nitrogen accumulation. However, it still maintained a high level of precision, with NRMSE values of 6.19–18.66% for plant nitrogen accumulation and 10.02–32.39% for grain nitrogen accumulation (Table 6). To date, there have been no reports on the use of this model to simulate plant and grain nitrogen accumulation in millet. Nevertheless, the model has been validated for its ability to simulate the impacts of nitrogen management on millet growth and yield [30,32]. This also indirectly confirms, to some extent, the model’s applicability in simulating plant nitrogen dynamics. Similar to the simulation of the LAI, aboveground biomass, and grain yield, the largest errors in simulating plant and grain nitrogen accumulation also occurred under low water and nitrogen treatments (Table 6). Simulating the plant nitrogen flux under low water and nitrogen conditions is also recognized as another significant challenge in the development of current crop models [47].

### 4.2. Simulation of Grain Protein Accumulation

Up until now, there have been no reports on the modeling of protein accumulation in millet grains. This study relied on the simulation of the dynamic changes in grain nitrogen accumulation and employed a nitrogen-to-protein conversion factor (NPF) to convert grain nitrogen accumulation to grain protein accumulation, thus enabling the simulation of grain protein accumulation in millet (Equation (1)). The method involving nitrogen dynamics simulation and NPF application is a standard procedure for estimating protein accumulation in cereals and has been validated in studies on wheat, maize, and rice [18,26,48]. The NRMSE values for grain protein accumulation under various water and nitrogen treatments in this study ranged from 3.06% to 26.22% (Figure 5), which is comparable to the grain protein simulation accuracy (NRMSE = 1.98–17.16%) reported in studies on maize and wheat [18,26,48]. According to the evaluation criteria established by Jamieson et al. [43], this NRMSE value falls within a satisfactory range. For the calculation of the NPF in our study, we adopted the approach from Li et al. [26] that considers how the plant’s water and nitrogen conditions affect the conversion of grain nitrogen to protein. The results of this study showed that across 18 water and nitrogen treatments in 2022 and 2023, the NPF varied within a range of 5.84 to 6.01 during the growing season, influenced by water and nitrogen stress. This considerable variability in the NPF highlights the importance of accounting for water and nitrogen effects in its calculation. The largest errors in simulating grain protein accumulation were observed under low water and nitrogen treatments. This result is not unexpected, given the high errors in simulating grain nitrogen accumulation under these treatments (Table 6).

### 4.3. Simulation of Grain Starch Accumulation

The simulation of grain starch accumulation is more challenging than that of protein simulation as starch synthesis involves more complex metabolic pathways. The existing methods for simulating starch accumulation in cereal grains are limited, and none of them involve millet. In our model for simulating starch accumulation in millet grains, the starch synthesis process was divided into two stages: the supply of carbon sources to the grain and the synthesis of starch from carbohydrates (primarily sucrose) within the grain. For the simulation of the carbon source supply to millet grains, we adopted the approach developed by Pan et al. [22] and Ji et al. [18]. The carbon source supply received by millet grains, which came partly from the translocation of the pre-anthesis-accumulated vegetative dry matter and the immediate photosynthetic products after anthesis, was indirectly calculated through the dynamic changes in the aboveground and vegetative dry matter, as simulated by the DSSAT model (Equations (9) and (10)). The method to simulate the carbon source supply to grains has already been used for simulating starch accumulation in the grains of wheat and rice [18,22,23]. Simulating the process of carbohydrate synthesis into starch within grains requires considering the effects of substrate concentration and enzyme activity on the synthesis rate, which includes factors affecting enzyme activity such as temperature, plant water and nitrogen status, and lifecycle stages [18,23]. Therefore, simulating starch synthesis from carbohydrates in grains is challenging, and in previous studies, this was predominantly approached using statistical models based on field experiment results, lacking a strong theoretical foundation [18,23,48]. In this study, the Michaelis–Menten equation was used to simulate the process of carbohydrate synthesis into starch within grains, taking into account the supply of carbon sources and factors affecting the activity of related enzymes. The Michaelis–Menten equation has been widely applied in biology, chemistry, and related fields, including the simulation of metabolic processes within plants [38,49,50]. The acceptable NRMSE (4.06–26.88%) in simulating starch accumulation in millet grains demonstrates the feasibility of applying the Michaelis–Menten equation for such simulations (Figure 6).

Moreover, the accumulation amounts of amylopectin and amylose in millet grains were simulated based on grain starch accumulation and the proportions of these two components within grain starch accumulation. This method was also employed in the study by Chen [23], which further validated its effectiveness. These starch components were well-simulated, with an NRMSE of 13.72% for grain amylose accumulation and 11.58% for grain amylopectin accumulation (Figure 7). Given that the ratio of amylopectin to amylose content in millet grains is a crucial factor determining eating quality, the ability of our developed model to simulate the accumulation of amylopectin and amylose in grains is significant for grain quality simulations [1,3].

### 4.4. Limitations of the Enhanced DSSAT-CERES-Millet Model

The enhanced DSSAT-CERES-Millet model has demonstrated its capability to dynamically simulate grain protein and starch accumulation in millet across varying irrigation and nitrogen conditions. Nonetheless, our enhanced model for starch accumulation in millet grains has some limitations. Although it accounts for the impact of water stress during the grain filling period on starch synthesis and accumulation, it does not consider water stress that occurs prior to this critical phase. However, according to the existing studies, cereals that experience drought prior to the grain filling stage may develop a “drought memory”, which can potentially regulate the expression of genes associated with this stage, thus impacting the starch synthesis capacity of the grains [51,52,53]. The limitation of the model may, to some extent, restrict its accuracy in simulating starch accumulation in millet grains. In our future work, we intend to improve the starch accumulation model for millet seeds by including the influence of water stress prior to the grain filling period on the synthesis rate calculations.

## 5. Conclusions

The DSSAT-CERES-Millet model excelled in simulating the accumulation of carbon and nitrogen within plants and their transfer to the grains, achieving the NRMSE values of 1.65–15.07% for the aboveground biomass, 9.71% for the grain yield, 6.19–18.66% for the plant nitrogen accumulation, and 10.02–32.39% for the grain nitrogen accumulation. This robust performance establishes it as a critical foundation for providing data support to the protein and starch simulation modules, enabling accurate simulation of grain protein and starch accumulation in millet. The protein and starch simulation modules we developed have proven to be effective. The protein simulation module uses a nitrogen-to-protein conversion coefficient to determine grain protein accumulation based on the grain nitrogen accumulation simulated by the DSSAT-CERES-Millet model. In the starch simulation module, the carbon source supply (carbohydrates) received by millet grains is calculated based on the simulated aboveground and vegetative dry matter by the DSSAT-CERES-Millet model, and starch synthesis is modeled using the Michaelis–Menten equation to convert carbohydrates into starch within the grains. By integrating the two newly developed modules, the enhanced DSSAT-CERES-Millet model now effectively models these components, with the NRMSE values of 3.06–26.22% for grain protein accumulation and 4.06–26.88% for grain starch accumulation. Furthermore, the model can accurately simulate the accumulation of amylopectin and amylose in grains at maturity, achieving an NRMSE of less than 14%. This robust model can guide irrigation and nitrogen management to improve millet grain quality.

## Figures and Tables

**Table 1:** Physical parameter values at different soil layers in the experimental field at the study site.

Soil Layer (cm)	Texture (%)			BD (g/cm^−3^)	Ks (cm/h^−1^)	θ_FC_ (cm^3^/cm^−3^)	θ_WP_ (cm^3^/cm^−3^)
	Sand	Silt	Clay				
0~20	43.2	45.1	11.7	1.54	1.21	0.23	0.10
20~40	47.7	26.8	25.5	1.58	0.52	0.25	0.12
40~60	30.6	53.2	16.2	1.48	0.76	0.28	0.14
60~80	37.3	54.3	8.4	1.32	1.23	0.32	0.15
80~100	26.3	63.1	10.6	1.42	0.98	0.32	0.15

Note: BD, bulk density; Ks, saturated hydraulic conductivity; θ_FC_, soil water content at field capacity; θ_WP_, soil water content at wilting point.

**Table 2:** Experimental treatments in the 2021, 2022, and 2023 growing seasons.

Years	Treatments	N application Amount (kg/ha^−1^)	Total Irrigation Amount (mm)	Irrigation Time (Amount) of Each Irrigation Event
2021	PSI-N180	180	100	4/25 (100 mm)
	FI-N180	180	280	4/25 (100 mm), 7/4 (60 mm), 8/4 (60 mm), 8/25 (60 mm)
2022	RF-N0	0	0	—
	RF-N180	180	0	—
	PSI-N0	0	100	4/21 (100 mm)
	PSI-N180	180	100	4/21 (100 mm)
	PVI-N0	0	220	4/21 (100 mm), 6/19 (60 mm), 7/18 (60 mm)
	PVI-N180	180	220	4/21 (100 mm), 6/19 (60 mm), 7/18 (60 mm)
	FI-N0	0	280	4/21 (100 mm), 6/19 (60 mm), 7/18 (60 mm), 8/3 (60 mm)
	FI-N90	90	280	4/21 (100 mm), 6/19 (60 mm), 7/18 (60 mm), 8/3 (60 mm)
	FI-N180	180	280	4/21 (100 mm), 6/19 (60 mm), 7/18 (60 mm), 8/3 (60 mm)
	FI-N270	270	280	4/21 (100 mm), 6/19 (60 mm), 7/18 (60 mm), 8/3 (60 mm)
2023	PSI-N0	0	100	4/24 (100 mm)
	PSI-N90	90	100	4/24 (100 mm)
	PSI-N180	180	100	4/24 (100 mm)
	PSI-N270	270	100	4/24 (100 mm)
	FI-N0	0	280	4/24 (100 mm), 6/10 (60 mm), 7/8 (60 mm), 8/27 (60 mm)
	FI-N90	90	280	4/24 (100 mm), 6/10 (60 mm), 7/8 (60 mm), 8/27 (60 mm)
	FI-N180	180	280	4/24 (100 mm), 6/10 (60 mm), 7/8 (60 mm), 8/27 (60 mm)
	FI-N270	270	280	4/24 (100 mm), 6/10 (60 mm), 7/8 (60 mm), 8/27 (60 mm)

**Table 3:** Calibration results of the parameters for models and modules related to simulating the grain protein and starch accumulation in foxtail millet.

Model/Module	Parameters	Description	Values
DSSAT–CERES-Millet model	P1	Thermal time from seedling emergence to the end of the juvenile phase, during which the plant is not responsive to changes in the photoperiod; GDD (°C d)	138.0
	P20	Critical photoperiod, or the longest day length at which development occurs at the maximum rate; at values higher than P20, the rate of development is reduced; hours	12.7
	P2R	Extent to which phasic development leading to panicle initiation is delayed for each hour increase in the photoperiod above P20; GDD (°C d)	32.0
	P5	Thermal time from the beginning of grain filling (3–4 days after anthesis) to physiological maturity; GDD (°C d)	485.0
	G1	Scaler for the relative leaf size	0.48
	G4	Scaler for partitioning of assimilates to the panicle (head)	3.35
	PHINT	Phyllochron interval; the interval between successive leaf tip appearances; GDD (°C d)	41.0
Grain protein simulation module	NPF_0_	The baseline value for the nitrogen-to-protein conversion factor	5.83
	δ	The correction factor for NPF_0_	0.18
Grain starch simulation module	IGSA_0_	The initial starch content in a single grain; mg per grain	0.1
	ISTR_m_	Maximum starch accumulation rate of a single grain; mg/day^−1^ per grain	1.2
	K_m_	Michaelis constant; mg per grain	0.7
	GDD_m_	The accumulated temperature from anthesis to peak grain synthetic amylase activity; GDD (°C d)	256
	γ	Sensitivity coefficient of amylase activity to the accumulation of growing degree days after anthesis	0.002
	α	Scaling factor that determines how the natural logarithm of growing degree days influences the ratio of amylose accumulation relative to total starch	0.4
	β	Correction factor that adjusts the baseline ratio of amylose to total starch independent of heat accumulation and nitrogen stress effects	2.1

**Table 4:** Statistical indices for evaluating the DSSAT-CERES-Millet model’s performance on the leaf area index and aboveground biomass under different water and nitrogen treatments from 2021 to 2023.

Year	Treatment	Leaf Area Index			Aboveground Biomass		
		MRD (%)	RMSE	NRMSE (%)	MRD (%)	RMSE (t/ha^−1^)	NRMSE (%)
2021	PSI-N180	−5.56	0.24	12.34	−2.60	0.57	9.94
	FI-N180	4.37	0.20	8.79	−6.03	0.43	7.08
2022	RF-N0	45.80	0.20	18.35	−14.30	0.47	15.07
	RF-N180	−22.42	0.24	16.69	7.55	0.24	8.23
	PSI-N0	−25.20	0.25	15.24	4.39	0.48	10.38
	PSI-N180	−21.60	0.35	14.02	−9.03	0.38	6.92
	PVI-N0	−22.34	0.27	13.74	9.60	0.30	6.11
	PVI-N180	−21.91	0.28	12.35	3.33	0.31	5.27
	FI-N0	−15.00	0.21	10.41	0.21	0.41	8.67
	FI-N90	−16.85	0.22	10.65	5.81	0.21	4.07
	FI-N180	−19.77	0.23	10.07	2.60	0.13	2.48
	FI-N270	−18.54	0.19	8.27	−2.87	0.10	1.65
2023	PSI-N0	−22.25	0.30	16.96	−22.23	0.94	14.34
	PSI-N90	−15.09	0.33	13.43	−28.45	1.06	12.87
	PSI-N180	−13.77	0.35	11.83	−24.47	0.87	9.32
	PSI-N270	−12.66	0.42	12.53	−25.41	1.10	10.52
	FI-N0	−15.38	0.28	14.06	−28.92	1.01	12.67
	FI-N90	−19.40	0.39	12.95	−26.46	1.27	11.71
	FI-N180	−15.97	0.42	11.21	−18.62	1.00	8.17
	FI-N270	−14.67	0.40	9.72	−13.86	0.88	6.26

Note: RF, PSI, PVI, and FI indicate different irrigation strategies. RF represents no irrigation or rain-fed treatment. In PSI, only a pre-sowing irrigation is adopted. In PVI, irrigation is conducted before sowing and at the vegetative growth stage of foxtail millet. In FI, irrigation is conducted before sowing and during the growing season to avoid water stress on foxtail millet. N0, N90, N180, and N270 are nitrogen application rates of 0, 90, 180, and 270 kg/ha^−1^, respectively. RMSE, NRMSE, and MRD stand for the root mean square error, the normalized RMSE, and the mean relative difference, respectively.

**Table 5:** Comparison between the measured phenology of foxtail millet and the simulated values from the DSSAT-CERES-Millet model under different water and nitrogen treatments from 2021 to 2023.

Year	Treatment	Anthesis (Days After Sowing)		Maturity (Days After Sowing)	
		Measured	Simulated	Measured	Simulated
2021	PSI-N180	101	100	150	155
	FI-N180	99	100	154	155
2022	RF-N0	100	98	140	145
	RF-N180	102	98	141	145
	PSI-N0	99	98	140	145
	PSI-N180	100	98	142	145
	PVI-N0	95	98	141	145
	PVI-N180	98	98	144	145
	FI-N0	96	98	140	145
	FI-N90	97	98	142	145
	FI-N180	98	98	145	145
	FI-N270	98	98	144	145
2023	PSI-N0	102	101	148	152
	PSI-N90	100	101	150	152
	PSI-N180	100	101	152	152
	PSI-N270	103	101	152	152
	FI-N0	98	101	151	152
	FI-N90	102	101	154	152
	FI-N180	100	101	153	152
	FI-N270	103	101	154	152

Note: RF, PSI, PVI, and FI indicate different irrigation strategies. RF represents no irrigation or rain-fed treatment. In PSI, only a pre-sowing irrigation is adopted. In PVI, irrigation is conducted before sowing and at the vegetative growth stage of foxtail millet. In FI, irrigation is conducted before sowing and during the growing season to avoid water stress on foxtail millet. N0, N90, N180, and N270 are nitrogen application rates of 0, 90, 180, and 270 kg/ha^−1^, respectively.

**Table 6:** Statistical indices for evaluating the DSSAT-CERES-Millet model’s performance on plant nitrogen accumulation and grain nitrogen accumulation of foxtail millet under the different water and nitrogen treatments in 2022 and 2023.

Year	Treatment	Plant Nitrogen Accumulation			Grain Nitrogen Accumulation		
		MRD (%)	RMSE (t/ha^−1^)	NRMSE (%)	MRD (%)	RMSE (t/ha^−1^)	NRMSE (%)
2022	RF-N0	14.55	6.13	18.66	−29.75	9.80	32.39
	RF-N180	−2.50	7.42	14.57	−19.16	6.81	18.47
	PSI-N0	5.64	4.98	13.04	−21.28	8.60	24.30
	PSI-N180	−0.80	7.43	12.61	−12.51	7.61	15.46
	PVI-N0	2.40	8.37	16.92	13.96	8.39	20.45
	PVI-N180	3.87	9.56	10.13	−10.11	10.65	13.03
	FI-N0	0.84	9.57	17.92	−24.81	16.71	27.19
	FI-N90	3.17	10.32	11.65	−16.51	21.76	21.66
	FI-N180	0.42	10.85	10.57	−11.15	11.83	12.58
	FI-N270	1.56	10.88	9.36	−7.21	11.27	11.61
2023	PSI-N0	9.53	5.31	9.37	−21.51	10.34	23.61
	PSI-N90	3.22	9.45	9.13	−15.85	15.55	20.37
	PSI-N180	8.35	13.65	11.55	−10.74	17.57	18.02
	PSI-N270	13.41	16.58	12.63	−11.59	15.21	15.69
	FI-N0	0.35	11.80	16.54	−25.12	17.67	28.29
	FI-N90	−5.47	7.39	6.24	−16.31	24.59	22.54
	FI-N180	0.84	16.35	11.02	−14.05	20.78	16.34
	FI-N270	3.15	10.75	6.19	−9.35	13.18	10.02

Note: RF, PSI, PVI, and FI indicate different irrigation strategies. RF represents no irrigation or rain-fed treatment. In PSI, only a pre-sowing irrigation is adopted. In PVI, irrigation is conducted before sowing and at the vegetative growth stage of foxtail millet. In FI, irrigation is conducted before sowing and during the growing season to avoid water stress on foxtail millet. N0, N90, N180, and N270 are nitrogen application rates of 0, 90, 180, and 270 kg/ha^−1^, respectively. RMSE, NRMSE, and MRD stand for the root mean square error, the normalized RMSE, and the mean relative difference, respectively.

## Data Availability

Data will be made available upon request.
